# Optimizing the programmatic deployment of the anti-malarials artemether-lumefantrine and dihydroartemisinin-piperaquine using pharmacological modelling

**DOI:** 10.1186/1475-2875-13-138

**Published:** 2014-04-07

**Authors:** Eva Maria Hodel, Katherine Kay, Daniel J Hayes, Dianne J Terlouw, Ian M Hastings

**Affiliations:** 1 Liverpool School of Tropical Medicine, Pembroke Place, Liverpool L3 5QA, UK; 2 Malawi-Liverpool-Wellcome Trust Clinical Research Programme, PO Box 30096, Chichiri, Blantyre 3, Malawi

**Keywords:** Falciparum malaria, Anti-malarials, Artemisinins, Dosing regimen, Mathematical model, Pharmacokinetics, Piperaquine, Lumefantrine, Drug resistance, Patient adherence, Medication adherence

## Abstract

**Background:**

Successful programmatic use of anti-malarials faces challenges that are not covered by standard drug development processes. The development of appropriate pragmatic dosing regimens for low-resource settings or community-based use is not formally regulated, even though these may alter factors which can substantially affect individual patient and population level outcome, such as drug exposure, patient adherence and the spread of drug resistance and can affect a drug’s reputation and its eventual therapeutic lifespan.

**Methods:**

An *in silico* pharmacological model of anti-malarial drug treatment with the pharmacokinetic/pharmacodynamic profiles of artemether-lumefantrine (AM-LF, Coartem®) and dihydroartemisinin-piperaquine (DHA-PPQ, Eurartesim®) was constructed to assess the potential impact of programmatic factors, including regionally optimized, age-based dosing regimens, poor patient adherence, food effects and drug resistance on treatment outcome at population level, and compared both drugs’ susceptibility to these factors.

**Results:**

Compared with DHA-PPQ, therapeutic effectiveness of AM-LF seems more robust to factors affecting drug exposure, such as age- instead of weight-based dosing or poor adherence. The model highlights the sub-optimally low ratio of DHA:PPQ which, in combination with the narrow therapeutic dose range of PPQ compared to DHA that drives the weight or age cut-offs, leaves DHA at a high risk of under-dosing.

**Conclusion:**

Pharmacological modelling of real-life scenarios can provide valuable supportive data and highlight modifiable determinants of therapeutic effectiveness that can help optimize the deployment of anti-malarials in control programmes.

## Background

Deploying new anti-malarial drugs is a three-stage process. Firstly, new drugs are developed through a research and development (R&D) pipeline (preclinical phase). Secondly, clinical studies (Phases I to III) demonstrate that the new drugs are effective and safe under controlled clinical conditions. Thirdly, the drugs are deployed under programmatic or real-life circumstances (post-marketing, Phase IV). The current regulatory drug development process provides limited guidance on the programmatic deployment of anti-malarials in low-resource settings and lacks consensus on the evidence base required to optimize real-life drug delivery. Undoubtedly, clinical trials will always remain the gold standard for experimental validation of drug effectiveness and safety and no policy-maker would ever solely rely on model predictions, but, for ethical reasons, certain clinical trials cannot reasonably be conducted, e g, trials estimating the effect of known sub-therapeutic doses, leaving an uncomfortable gap in our knowledge that might be at least partly bridged by the output generated using modelling approaches.

Pharmacological drug treatment models can be constructed to assess dosing accuracy and impact at patient and population level and could constitute a strong support role in the policy decision-making process in regard to pharmacological aspects. Their potential has been highlighted both for the development of paediatric formulations for the ‘Essential Medicines List’ [1] and to analyse the likely effects of drugs on malaria throughout a global eradication campaign [2]. Accurate simulations can rapidly investigate the consequences of varied drug deployment strategies and help identify optimal dosage levels, frequency and duration. Moreover, they can be used to investigate real-life situations that cannot be field tested, such as anticipated sub- or supra-therapeutic dosing in patients around the cut-off points of dosing bands or the impact of poor patient adherence on treatment outcome, and the potential lessons for programmatic delivery strategies and the associated monitoring and evaluation. Similarly, they can be used to predict susceptibility to, and efficacy of, anti-malarials at a time of developing drug tolerance or resistance and its subsequent spread through the population [3]. Important factors that determine treatment success in programmatic settings are linked to drug exposure related to dosing regimens, treatment adherence and drug resistance. Others include drug quality, drug interactions, poor or erratic absorption and misdiagnosis [4].

Decades of work on malaria models have resulted in numerous models of its epidemiology, and the genetics of drug resistance, but fewer than a dozen published models specifically focus on anti-malarial drug treatment [3,5-13]. Drug treatment models for malaria are based on mathematical pharmacokinetic/pharmacodynamic (PK/PD) models describing the effect of antimicrobial drugs (recently reviewed by Czock & Keller [14]). In the case of malaria, these models use differential equations to track parasite growth (the parasite submodel), the effect of anti-malarial drugs (the anti-malarial submodel), and changing drug concentrations (the pharmacokinetic submodel) [14]. The differential equations are then used to describe the dynamics of a parasite population in the presence of anti-malarial drug treatment. Existing PK/PD models either investigate only monotherapy [5-7,9-11], do not model the artemisinin-based combination therapy (ACT) drug components individually [8] or are not able to reliably replicate the results of clinical trials [12]. None of the PK/PD models specifically aim to simulate programmatic settings where, e g, (un-)intended alterations of intake dose or drug resistance can jeopardize drug effectiveness and safety. Models to investigate these real-life challenges are therefore required to address substantial gaps in our evidence base, and must be able to (i) allow investigation of the effectiveness of several drugs co-administered as ACT; (ii) allow changes in PK/PD parameters of individual ACT drug components (e g, increasing resistance, genetic variation in drug elimination); and, (iii) reliably reproduce data from clinical trials.

This study presents a novel PK/PD model that integrates two existing models to simulate the real-life population level effectiveness of artemether-lumefantrine (AM-LF) and dihydroartemisinin-piperaquine (DHA-PPQ) against background scenarios of varying age and weight-based dosing regimens and levels of drug adherence, some of the many challenges faced when deploying anti-malarials in programmatic settings.

## Methods

**Figure 1:**
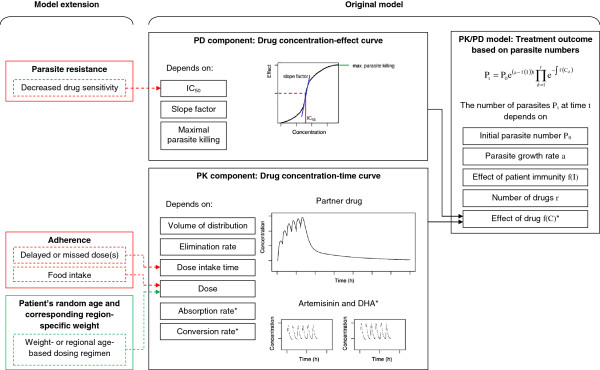
**Overview showing the original PK/PD model (black boxes), the extension to allow for region specific dosing by weight or age (green box) and the different treatment scenarios simulated (red boxes).** * If artesunate or artemether are given, the model accounts for their absorption and conversion into the active metabolite dihydroartemisinin (DHA). The model then updates parasite numbers in the next time step using only the drug with the larger effect, i.e. either the parent drug or DHA.

In order to predict drug effectiveness at the individual and population level and to investigate the effects of decreased parasite drug susceptibility to treatment and poor patient adherence an already existing, validated, *in silico* simulation of anti-malarial drug action (hereafter referred to as the ‘original model’) [3,13] was extended. While the original model simulated treatment outcomes based on variation in individual patient PK/PD parameters it only assumed each patient received the ‘correct’ mg/kg dose. As one of the main interests of this study was the comparison of weight- and age-based dosing regional specific weight-for-age tables were integrated to assign realistic body weights across all ages of the population [15]. This allowed simulated patients to vary in age and weight so that patient dosages (in mg/kg) would vary according to proposed age-based and weight-based dosing regimens. The model was implemented in the statistical software package R version 3.0.1 [16]. A schematic representation of the model targeted at an audience with a rather non-modelling background is presented in Figure 1. The following sections are mainly directed at readers with particular interest in the mathematical and technical details of the drug treatment model, its calibration and validation.

### Original drug treatment model

This study adapted the validated PK/PD model of Winter & Hastings [13] which predicts treatment outcome in adults based on individual human and infection parameters including (i) varied patient PK, i.e. volume of distribution and elimination rate, (ii) varied parasite drug sensitivity (PD), i e, half-maximum inhibitory concentration (IC_50_), maximal parasite-killing rate constant and slope factor, and (iii) varied parasite densities at time of treatment. The additional absorption and conversion phases for the artemisinins, and the parameter specific estimates of variation around a mean as described in Kay & Hastings [3] were included (Table 1, for details and references see [13] and supplementary material in [3]). Parameters were assumed to be normally distributed when CV <50% and log-normally distributed for CV ≥50% [3]. No parameter specific CV estimates could be found for the first-order rate constant of parasite killing (V_max_) and the slope factor (n) and so a default CV of 30% was assumed [13]. For this study the mean volume of distribution (Vd) for AM was changed from central Vd to the steady-state Vd (i.e. central plus peripheral Vd) reported by [17] so simulations reproduce cure rates from field data for AM monotherapy [18-20] and AM-LF combination therapy [21]. The mean Vd for DHA was changed to that reported by Giao & de Vries [22] to reproduce cure rates from field data for AM monotherapy [18-20] and AM-LF combination therapy [21]. The mean Vd for DHA was changed to the DHA metabolite Vd measured following artesunate treatment (for details and references see supplementary material in [3]) to reproduce cure rates from field data for DHA monotherapy [23] and DHA-PPQ combination therapy in adults and children [24].

**Table 1:** Anti-malarial drug parameters for artemether-lumefantrine (AM-LF) and dihydroartemisinin-piperaquine (DHA-PPQ) combination therapies

**Drug parameter**	**Artemether + lumefantrine**			**Dihydroartemisinin + piperaquine**	
	**AM**	**DHA**	**LF**	**DHA**	**PPQ**
Vd (L/kg)	46.6 (82)	15 (48)	21 (263)	1.49 (48)	150 (42)
x (/day)	23.98 (68)	–	–	–	–
z (/day)	11.97 (65)	–	–	–	–
k (/day)	–	44.15 (23)	0.16 (5)	19.8 (23)	0.03 (54)
IC_50_ (mg/L)	0.0023 (79)	0.009 (117)	0.032 (102)	0.009 (117)	0.088 (30)
V_max_	27.6	27.6	3.45	27.6	3.45
n	4	4	4	4	6

The means with associated variation (i e, coefficient of variation, CV) are given in brackets.

Vd: volume of distribution; x: absorption rate constant; z: conversion rate constant; k: elimination rate constant; IC_50_: Concentration producing half the desired effect; V_max_: first-order rate constant of parasite killing; n: slope factor.

The number of parasites present at the start of treatment *P*_*0*_ was chosen randomly from a uniform distribution of between 10^10^ and 10^12^. The mean maximal parasite growth rate *a* was equal to 1.15 with a 30% variation around the mean. Artemisinin derivatives were assumed to share the same mechanism of action and so used only the derivative that achieves the higher drug effect on the parasites in a time step were used [3]. All patients were assumed to be malaria naïve and have no acquired immunity.

In brief, this pharmacological model uses a standard differential equation to find a mathematical description for the rate of change in total parasite growth and death rates

(1) $$\frac{\mathrm{dP}}{\mathrm{dt}}=P\cdot \left(a-f\left(I\right)-f\left(C\right)\right)$$

where *P* is the number of parasites in the infection, *t* is time after treatment (days), *a* is the parasite growth rate (per day), *f(C)* represents the drug-dependent rate of parasite killing which depends on the drug concentration *C*, and *f(I)* the killing resulting from the hosts background immunity. As anti-malarial drugs are now typically deployed as combination therapies and as each drug may affect parasites in its unconverted and/or converted forms, predicting the changing numbers of parasites requires an expansion of Equation 1

(2) $$\frac{\mathrm{dP}}{\mathrm{dt}}=P\cdot \left(a-f\left(I\right)-\sum_{d=1}^{r}f\left(C_{d}\right)\right)$$

where *r* is the number of drugs, the drug effect *f(C*_*d*_*)* is the effect of each drug, *d*. Note that each active entity was regarded as a distinct ‘drug’. Reasons for not including synergism and cross-resistance between drugs in a combination are discussed in detail elsewhere [13]. For example artemether-lumefantrine (AM-LF) is three drugs LF, AM (unconverted) and its active metabolite dihydroartemisinin (DHA). Integrating Equation 2 using the separation-of-variables technique (for further details see step-wise transition from Equation 10 to Equation 16 in Kay & Hastings [3]) allows to predict the number of parasites at any time, *t*, after treatment with any number of drugs, as

(3) $$P_{t}=P_{0}e^{\left(a-f\left(I\right)\right)t}\prod_{d=1}^{r}e^{-\int f\left(C_{d}\right)}$$

Details on how the parameters described in Table 1 were selected and used to calculate *f(C*_*d*_*)* of each drug, *d*, can be found in [3].

The original model by Winter & Hastings [13] calculates the parasite numbers every 12 h during the first 7 days to allow for multiple-dose regimens and then every 24 h. In order to allow for 8-hourly (AM-LF first day) and 12-hourly dosing the time steps were decreased to 4 h for the first 7 days, leading to slightly different results for AM because of the parasite kill *versus* time profile (i e, Figure S2 in supplementary material in [3] shows that the concentration of AM is much higher at 4 h than 12 h). As the switch from 12 h to 4 h time steps for the first 7 days did not significantly alter treatment outcome, even smaller time steps (e g, 1 h) were not considered in order to speed up simulations.

The original model was only validated to predict treatment outcome in adults. In order to validate the model for children maximal concentrations (c_max_) and times to maximal concentration (t_max_) were calculated using the same PK parameters as in adults (Table 1) without allometric scaling for children of the same weight as the average weight of the respective children included in studies on AM and its metabolite DHA in combination with the partner drug LF [3] and piperaquine (PPQ) when given with DHA [25-28]. Due to the lack of pharmacokinetic data for DHA given in combination with PPQ only in children validation of the model for DHA was not possible. It was therefore assumed DHA concentrations when given with PPQ are comparable to DHA concentrations observed in AM-LF treatment where DHA is the active metabolite of AM. Overlap of predicted c_max_ and t_max_ and field observations was found to be good and consequently the same PK parameters for all age groups were used (Table 1).

### Integration of weight-for-age distribution of malaria endemic regions

Regional, weight-for-age references representative of the population in malaria-endemic countries in Africa, Asia and Latin America were integrated. These references were based on a modelling method recently developed to allow the generation of statistically robust weight-for-age references from multi-source data [29]. The use of these weight-for-age references allowed to assign realistic region-specific weights for patients between 6 months and 25 years of age. Each hypothetical individual was assigned a random age (in monthly increments) between 6 months and 25 years and a random weight-for-age percentile from the regional reference. When using weight-based dosing, sampled individuals below 5 kg were excluded from the analysis and replaced by a new random sample record.

The weight-for-age references were also used to generate optimised age-based regimens for AM-LF and DHA-PPQ [15]. The regimens used in this publication are described in Table 2. Note that for DHA-PPQ the manufacturer recommends a weight-based dosing regimen with 6 bands (the highest band for individuals weighing 75–100 kg). When employing regional growth reference to optimise the predicted number of patients receiving doses within the therapeutic range only 4 or 5 bands are employed.

**Table 2:** The weight- and age-based dosing regimens investigated for fixed-dose artemether-lumefantrine (AM-LF) and dihydroartemisinin-piperaquine (DHA-PPQ)

	**Weight category**	**Age category**				**Number of tablets**
	**Drug**	**Global**	**Africa**	**Latin America**	**Asia**	
AM-LF	5–14 kg^1^	6 m–3 yrs	6 m–3 yrs	6 m–2 yrs	6 m–3 yrs	1 × 20/120 mg^2^
	15–24 kg^1^	4–10 yrs	4–9 yrs	3–7 yrs	4–11 yrs	2 × 20/120 mg^2^
	25–34 kg^1^	11–14 yrs	10–12 yrs	8–10 yrs	12–14 yrs	3 × 20/120 mg^2^
	35 + kg^1^	≥15 yrs	≥13 yrs	≥11 yrs	≥15 yrs	4 × 20/120 mg^2^
DHA-PPQ	5– < 7 kg^3^	6 m–2 yrs	6 m–1 yr	6 m–1 yr	6 m–2 yrs	0.5 × 20/160 mg^4^
	7– < 13 kg^3^	3–9 yrs	2–8 yrs	2–6 yrs	3–10 yrs	1 × 20/160 mg^4^
	13– < 24 kg^3^	10–14 yrs	9–14 yrs	7–11 yrs	11–15 yrs	1 × 40/320 mg^4^
	24– < 36 kg^3^	≥15 yrs	15–19 yrs	12–16 yrs	≥16 yrs	2 × 40/320 mg^4^
	36– < 75 kg^3^	–	≥20 yrs	≥17 yrs	–	3 × 40/320 mg^4^
	75–100 kg^3^	–	–	–	–	4 × 40/320 mg^4^

^1^As provided in the World Health Organization *Guidelines for the treatment of malaria *[30]. ^2^twice daily dose for 3 days (at 0 h, 8 h, 24 h, 36 h, 48 h, 60 h); ^3^ as provided by the manufacturer [31]; ^4^ daily dose for 3 days (at 0 h, 24 h, 48 h).

### Sample size and follow-up time

In a pilot analysis the 95% confidence intervals (CIs) of the percentage of individuals that had cleared the parasites were compared by running the models with 10,000 and 1 million individuals. Larger samples had narrower CIs but the lower and upper boundaries of the CIs differed by less than 2% points (e g, 91% *versus* 93%) and it was therefore decided to sample only 10,000 individuals in order to decrease computing time. The post-treatment follow-up duration of 63 days was chosen in accordance with the WHO recommendation for the minimal post-treatment follow-up duration for long half-life drugs in clinical trials [32].

### Treatment scenarios

Individuals were either dosed according to regional age-bands or global weight-bands. Then the performance of the two dosing strategies was tested in different programmatic scenarios, such as poor patient adherence to treatment, the effect of food intake and the impact on parasite resistance expressed as IC_50_.

For illustrative purposes two examples of ACT were chosen: (i) AM-LF which has become the most widely recommended first-line regimen for the treatment of uncomplicated falciparum malaria in Africa [33], and, (ii) DHA-PPQ which is being considered a highly promising drug for global deployment due to its simple once-a-day dosing and extended post-treatment prophylactic effect [34-38]. The weight- and age-based regimens used in this publication are presented in Table 1. The methodology is sufficiently flexible that other proposed regimens could be evaluated, and compared. It is of note that a separate modelling approach, which used the same regional specific weight-for-age tables mentioned above, has been employed to predict the optimized age-based regimens for ACT for case management of uncomplicated malaria [15] that were used in this study. As neither the manufacturer of Eurartesim®, the only pharmaceutical DHA-PPQ product that obtained marketing authorization from a stringent regulatory authority, nor the current WHO Guidelines for the Treatment of Malaria [30] give recommendations on global age-based regimens, Hayes *et al.* optimized age-banding thresholds for each drug for each of the three global malaria-endemic regions (Asia, Africa and Latin America) to result in the highest possible number of individuals receiving a mg/kg dose within the therapeutic range currently recommended by WHO [30].

## Results

Treatment outcome at 63 days was categorized as either (i) all parasites cleared (<1 parasite); (ii) parasites still present but below the microscopic limit of detection (LoD) of 10^8^ parasites and might subsequently either clear or recrudesce; (iii) drug failures, ‘recrudescences’ in the malaria jargon, above LoD; or, (iv) parasites never cleared, i e, above LoD during entire post-treatment period.

### Fully adherent patients

**Figure 2:**
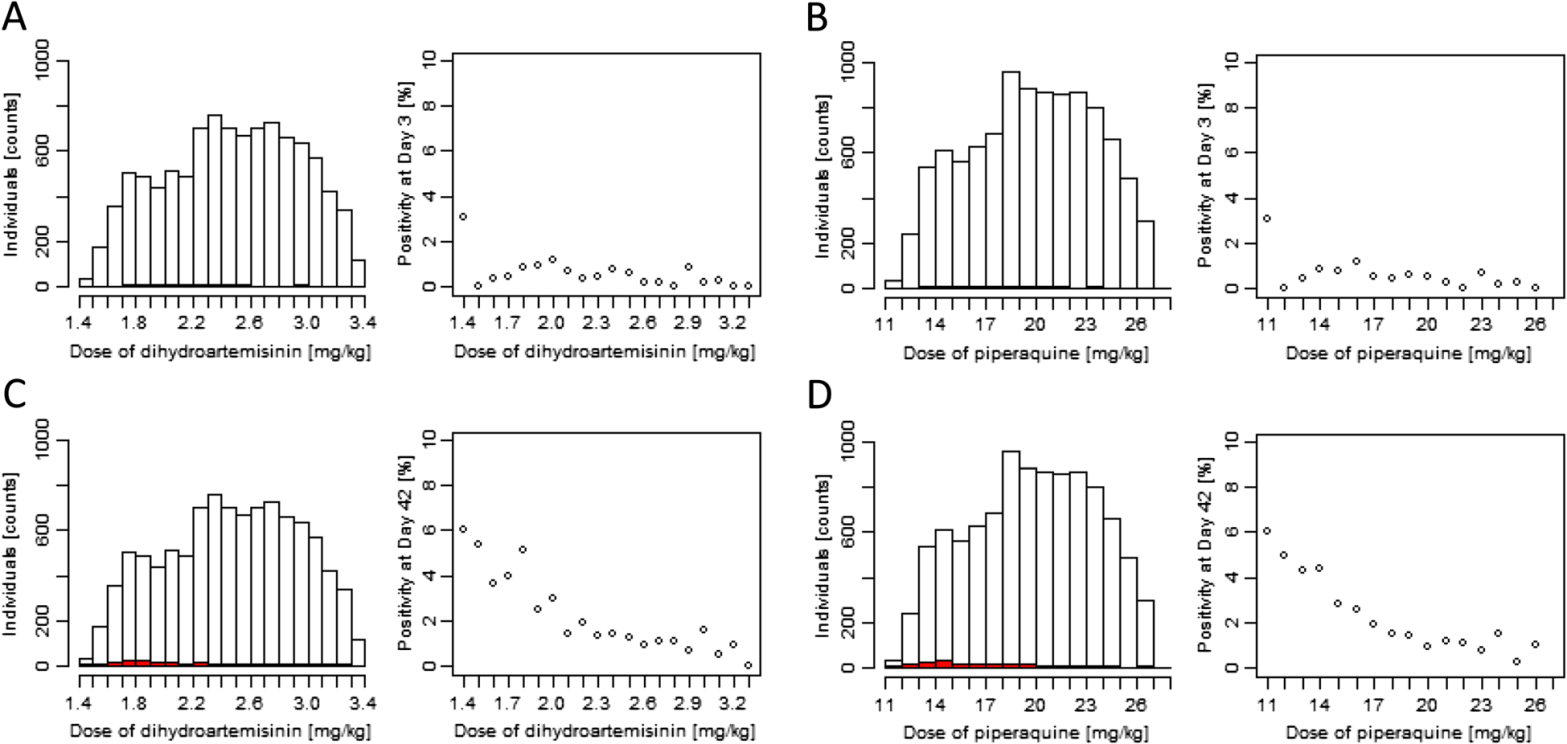
**Predicted parasite positivity at Day 3 and 42 in a global population of 10,000 individuals dosed by weight with dihydroartemisinin-piperaquine (DHA-PPQ).** Panels show predicted parasite positivity for DHA and PPQ at Day 3 (**(A)** and **(B)**, respectively) and at Day 42 (**(C)** and **(D)**, respectively). Left of panels: Total numbers of individuals per mg/kg dosing band (white) and individuals that were parasiaemic (i e, parasite number > 10^8^) (red). Right of panels: Numbers expressed as a percentage.

Each individual was assigned a full course of AM-LF (twice daily over three days at 0, 8, 24, 36, 48 and 60 hours) or DHA-PPQ (once daily over three days at 0, 24 and 48 hours) each either dosed by age or weight. The percentage of individuals in each dosing band with a mg/kg dose below or above the therapeutic dose range are summarized in Tables 3 and 4. The percentage of individuals with mg/kg doses below the WHO-defined therapeutic dose ranges of DHA and PPQ was calculated for comparison with a recently published pooled analysis by the WorldWide Antimalarial Resistance Network (WWARN) on DHA-PPQ dosing [39]. For a global population the weight-based regimen in our study would under-dose 44.13% of children under the age of 5 years with both DHA and PPQ (compared to 14.42% in older children and adults or 19.89% in the overall population). Individuals with low DHA-PPQ doses were more likely to be parasitaemic (i e, showing more than 10^8^ parasite) at Day 42 (Figure 2).

**Table 3:** **Percentage of individuals with mg/kg doses below or above the therapeutic dose range (according to **[30]**) of 1.4–4 mg/kg artemether (AM) and 10–16 mg/kg lumefantrine (LF) in each weight- and age-based dosing band**

**Number of tablets**		**Age-based dosing regimen**				**Weight-based dosing regimen**			
		**Global**	**Africa**	**Asia**	**Latin America**	**Global**	**Africa**	**Asia**	**Latin America**
1 × 20/120 mg	n	1448	1448	1448	1063	2135	1848	2204	1455
	AM below	6.28%	9.32%	4.97%	5.93%	11.94%	11.53%	10.84%	10.45%
	AM above	0.07%	0.07%	0.21%	0.00%	0.00%	0.00%	0.00%	0.00%
	LF below	26.66%	31.63%	23.90%	27.85%	46.60%	43.99%	45.78%	43.44%
	LF above	11.05%	10.64%	11.53%	6.49%	7.35%	8.17%	8.35%	4.74%
2 × 20/120 mg	n	2781	2403	3216	2019	2497	2231	2585	1857
	AM below	2.45%	2.25%	2.52%	1.78%	0.00%	0.00%	0.00%	0.00%
	AM above	0.22%	0.17%	0.19%	0.00%	0.00%	0.00%	0.00%	0.00%
	LF below	8.45%	10.78%	8.86%	9.26%	7.13%	7.08%	6.42%	6.89%
	LF above	26.65%	20.06%	25.40%	21.15%	0.00%	0.00%	0.00%	0.00%
3 × 20/120 mg	n	1706	1270	1271	1147	1578	1307	1659	1206
	AM below	4.98%	1.81%	2.28%	3.05%	0.00%	0.00%	0.00%	0.00%
	AM above	0.18%	0.00%	0.00%	0.00%	0.00%	0.00%	0.00%	0.00%
	LF below	13.07%	9.06%	9.76%	10.99%	0.00%	0.00%	0.00%	0.00%
	LF above	17.76%	15.04%	11.49%	15.69%	0.00%	0.00%	0.00%	0.00%
4 × 20/120 mg	n	4065	4879	4065	5771	3790	4614	3552	5482
	AM below	8.83%	24.78%	2.44%	34.21%	9.55%	26.16%	2.48%	35.94%
	AM above	0.00%	0.00%	0.00%	0.00%	0.00%	0.00%	0.00%	0.00%
	LF below	26.17%	57.45%	14.42%	66.14%	28.94%	60.71%	17.23%	69.66%
	LF above	1.62%	2.46%	2.07%	2.41%	0.00%	0.00%	0.00%	0.00%

n: number of individuals in respective dosing band.

**Table 4:** **Percentage of individuals with mg/kg doses below or above the therapeutic dose range (according to **[30]**) of 2–10 mg/kg dihydroartemisinin (DHA) and 16–26 mg/kg piperaquine (PPQ) in each weight- and age-based dosing band**

**Number of tablets**		**Age-based dosing regimen**				**Weight-based dosing regimen**			
		**Global**	**Africa**	**Asia**	**Latin America**	**Global**	**Africa**	**Asia**	**Latin America**
0.5 × 20/160 mg	n	1037	657	1037	657	99	89	105	35
	DHA below	99.04%	98.48%	99.04%	99.85%	100.00%	100.00%	100.00%	100.00%
	DHA above	0.00%	0.00%	0.00%	0.00%	0.00%	0.00%	0.00%	0.00%
	PPQ below	99.04%	98.48%	99.04%	99.85%	100.00%	100.00%	100.00%	100.00%
	PPQ above	0.00%	0.00%	0.00%	0.00%	0.00%	0.00%	0.00%	0.00%
1 × 20/160 mg	n	2836	2825	3222	2037	1343	1204	1401	1003
	DHA below	98.55%	95.72%	98.70%	98.63%	59.20%	57.97%	59.31%	61.32%
	DHA above	0.00%	0.00%	0.00%	0.00%	0.00%	0.00%	0.00%	0.00%
	PPQ below	98.55%	95.72%	98.70%	98.63%	59.20%	57.97%	59.31%	61.32%
	PPQ above	0.00%	0.00%	0.00%	0.00%	0.00%	0.00%	0.00%	0.00%
1 × 40/320 mg	n	2005	2396	2053	1973	2946	2499	3063	2106
	DHA below	87.23%	94.49%	93.18%	93.82%	29.33%	32.53%	28.73%	31.43%
	DHA above	0.00%	0.00%	0.00%	0.00%	0.00%	0.00%	0.00%	0.00%
	PPQ below	87.23%	94.49%	93.18%	93.82%	29.33%	32.53%	28.73%	31.43%
	PPQ above	0.00%	0.00%	0.00%	0.00%	0.00%	0.00%	0.00%	0.00%
2 × 40/320 mg	n	4122	2104	3688	2054	1908	1606	1990	1462
	DHA below	64.07%	86.98%	60.93%	77.07%	0.00%	0.00%	0.00%	0.00%
	DHA above	0.00%	0.00%	0.00%	0.00%	0.00%	0.00%	0.00%	0.00%
	PPQ below	64.07%	86.98%	60.93%	77.07%	0.00%	0.00%	0.00%	0.00%
	PPQ above	0.05%	0.00%	0.00%	0.15%	5.56%	6.79%	5.48%	5.54%
3 × 40/320 mg	n	0	2018	0	3279	3686	4491	3441	5174
	DHA below	–	31.27%	–	39.92%	6.27%	16.90%	1.08%	24.70%
	DHA above	–	0.00%	–	0.00%	0.00%	0.00%	0.00%	0.00%
	PPQ below	–	31.27%	–	39.92%	6.27%	16.90%	1.08%	24.70%
	PPQ above	–	0.05%	–	0.00%	5.18%	2.58%	6.02%	2.11%
4 × 40/320 mg	n	0	0	0	0	18	111	0	220
	DHA below	–	–	–	–	0.00%	48.65%	–	45.00%
	DHA above	–	–	–	–	0.00%	0.00%	–	0.00%
	PPQ below	–	–	–	–	0.00%	48.65%	–	45.00%
	PPQ above	–	–	–	–	0.00%	0.00%	–	0.00%

n: number of individuals in respective dosing band.

Cure rates for both treatments were very high across ages in fully adherent patients (Tables 5 and 6). When it was assumed that parasites would be fully sensitive to treatment, the proportion of individuals that cleared all parasites after treatment with AM-LF was predicted to be more than 99% regardless of region and dosing regimen (Table 5). Parasites in individuals treated with a full treatment of DHA-PPQ were cleared in about 91% of cases for the age-based dosing regimen and 97% for the weight-based dosing regimen (Table 6).

**Table 5:** **Predicted artemether-lumefantrine (AM-LF) treatment outcome (in %) in populations of 10,000 individuals at 63 days follow-up for different treatment scenarios group by dosing regimen (according to Table **2**) and geographical region**

**Treatment scenario**	**Treatment outcome**	**Age-based dosing regimen**				**Weight-based dosing regimen**			
		**Global**	**Africa**	**Asia**	**Latin America**	**Global**	**Africa**	**Asia**	**Latin America**
Full course, i e, twice daily over three days at 0, 8, 24, 36, 48 and 60 hours	All parasites cleared	99.83	99.81	99.83	99.81	99.83	99.82	99.85	99.82
	Parasites below LoD	0.01	0.02	0.01	0.02	0.01	0.02	0.01	0.02
	Recrudescence	0.15	0.16	0.15	0.16	0.15	0.15	0.13	0.15
	Parasites always above LoD	0.01	0.01	0.01	0.01	0.01	0.01	0.01	0.01
Fifth dose missed	All parasites cleared	99.78	99.75	99.77	99.68	99.79	99.75	99.78	99.68
	Parasites below LoD	0.01	0.02	0.01	0.02	0.01	0.02	0.01	0.02
	Recrudescence	0.20	0.22	0.21	0.29	0.19	0.22	0.20	0.29
	Parasites always above LoD	0.01	0.01	0.01	0.01	0.01	0.01	0.01	0.01
Sixth dose missed	All parasites cleared	99.78	99.75	99.77	99.68	99.79	99.75	99.78	99.68
	Parasites below LoD	0.01	0.02	0.01	0.02	0.01	0.02	0.01	0.02
	Recrudescence	0.20	0.22	0.21	0.29	0.19	0.22	0.20	0.29
	Parasites always above LoD	0.01	0.01	0.01	0.01	0.01	0.01	0.01	0.01
Fifth and sixth dose missed	All parasites cleared	99.52	99.39	99.55	99.24	99.56	99.4	99.53	99.24
	Parasites below LoD	0.02	0.01	0.02	0.02	0.02	0.01	0.02	0.02
	Recrudescence	0.45	0.58	0.42	0.72	0.41	0.58	0.44	0.72
	Parasites always above LoD	0.01	0.02	0.01	0.02	0.01	0.01	0.01	0.02
Third, fourth, fifth and sixth dose missed	All parasites cleared	95.33	96.56	96.93	96.35	95.07	94.53	95.34	94.21
	Parasites below LoD	0.01	0.01	0.03	0.02	0.01	0.01	0.01	0.00
	Recrudescence	4.50	3.30	2.92	3.48	4.75	5.21	4.50	5.53
	Parasites always above LoD	0.16	0.13	0.12	0.15	0.17	0.25	0.15	0.26
Third, fourth, fifth and sixth dose delayed by 12 hours	All parasites cleared	99.83	99.82	99.83	99.81	99.83	99.83	99.85	99.82
	Parasites below LoD	0.01	0.02	0.01	0.02	0.01	0.02	0.01	0.02
	Recrudescence	0.15	0.15	0.15	0.16	0.15	0.14	0.13	0.15
	Parasites always above LoD	0.01	0.01	0.01	0.01	0.01	0.01	0.01	0.01
Increased IC_50_ by 50-fold for LF and 10-fold for AM and DHA	All parasites cleared	76.85	73.89	77.62	72.5	75.1	72.99	76.17	71.49
	Parasites below LoD	0.02	0.02	0.02	0.00	0.02	0.01	0.01	0.01
	Recrudescence	15.49	17.02	15.15	17.7	16.35	17.4	15.72	18.18
	Parasites always above LoD	7.64	9.07	7.21	9.80	8.53	9.60	8.10	10.32
Administered without food, i e, 50% lower dose for LF	All parasites cleared	99.82	99.81	99.83	99.81	99.82	99.8	99.85	99.75
	Parasites below LoD	0.01	0.02	0.01	0.02	0.01	0.02	0.01	0.02
	Recrudescence	0.16	0.16	0.15	0.16	0.16	0.16	0.13	0.21
	Parasites always above LoD	0.01	0.01	0.01	0.01	0.01	0.02	0.01	0.02

DHA: dihydroartemisinin; IC_50_: Concentration producing half the desired effect; LoD: limit of detection (10^8^ parasites).

**Table 6:** **Predicted dihydroartemisinin-piperaquine (DHA-PPQ) treatment outcome (in %) in populations of 10,000 individuals at 63 days follow-up for different treatment scenarios group by dosing regimen (according to Table **2**) and geographical region**

**Treatment scenario**	**Treatment outcome**	**Age-based dosing regimen**				**Weight-based dosing regimen**			
		**Global**	**Africa**	**Asia**	**Latin America**	**Global**	**Africa**	**Asia**	**Latin America**
Full course, i e, once daily over three days at 0, 24 and 48 hours	All parasites cleared	90.53	91.03	90.33	92.21	97.73	97.49	97.78	97.36
	Parasites below LoD	0.40	0.47	0.36	0.40	0.41	0.38	0.40	0.46
	Recrudescence	8.50	7.93	8.67	6.98	1.82	2.10	1.80	2.11
	Parasites always above LoD	0.57	0.57	0.64	0.41	0.04	0.03	0.02	0.07
Second dose missed	All parasites cleared	69.24	69.75	68.32	72.70	90.68	89.7	91.06	88.8
	Parasites below LoD	0.50	0.55	0.54	0.45	0.52	0.60	0.49	0.65
	Recrudescence	24.01	23.73	24.68	21.92	8.13	8.91	7.76	9.59
	Parasites always above LoD	6.25	5.97	6.46	4.93	0.67	0.79	0.69	0.96
Third dose missed	All parasites cleared	70.46	71.16	69.83	74.02	91.2	90.34	91.61	89.48
	Parasites below LoD	0.50	0.50	0.48	0.44	0.48	0.58	0.45	0.62
	Recrudescence	24.28	23.76	24.75	22.01	7.77	8.45	7.45	9.16
	Parasites always above LoD	4.76	4.58	4.94	3.53	0.55	0.63	0.49	0.74
Second and third dose missed	All parasites cleared	27.06	27.64	26.04	30.01	51.90	49.55	52.23	48.77
	Parasites below LoD	0.32	0.31	0.32	0.34	0.61	0.47	0.59	0.44
	Recrudescence	31.06	31.02	31.37	31.53	31.25	31.91	31.42	31.63
	Parasites always above LoD	41.56	41.03	42.27	38.12	16.24	18.07	15.76	19.16
Second and third dose delayed by 24 hours	All parasites cleared	89.80	90.18	89.48	91.55	97.62	97.36	97.65	97.19
	Parasites below LoD	0.44	0.47	0.41	0.40	0.41	0.40	0.44	0.47
	Recrudescence	8.96	8.54	9.29	7.42	1.93	2.20	1.88	2.24
	Parasites always above LoD	0.80	0.81	0.82	0.63	0.04	0.04	0.03	0.10

LoD: limit of detection (10^8^ parasites).

**Figure 3:**
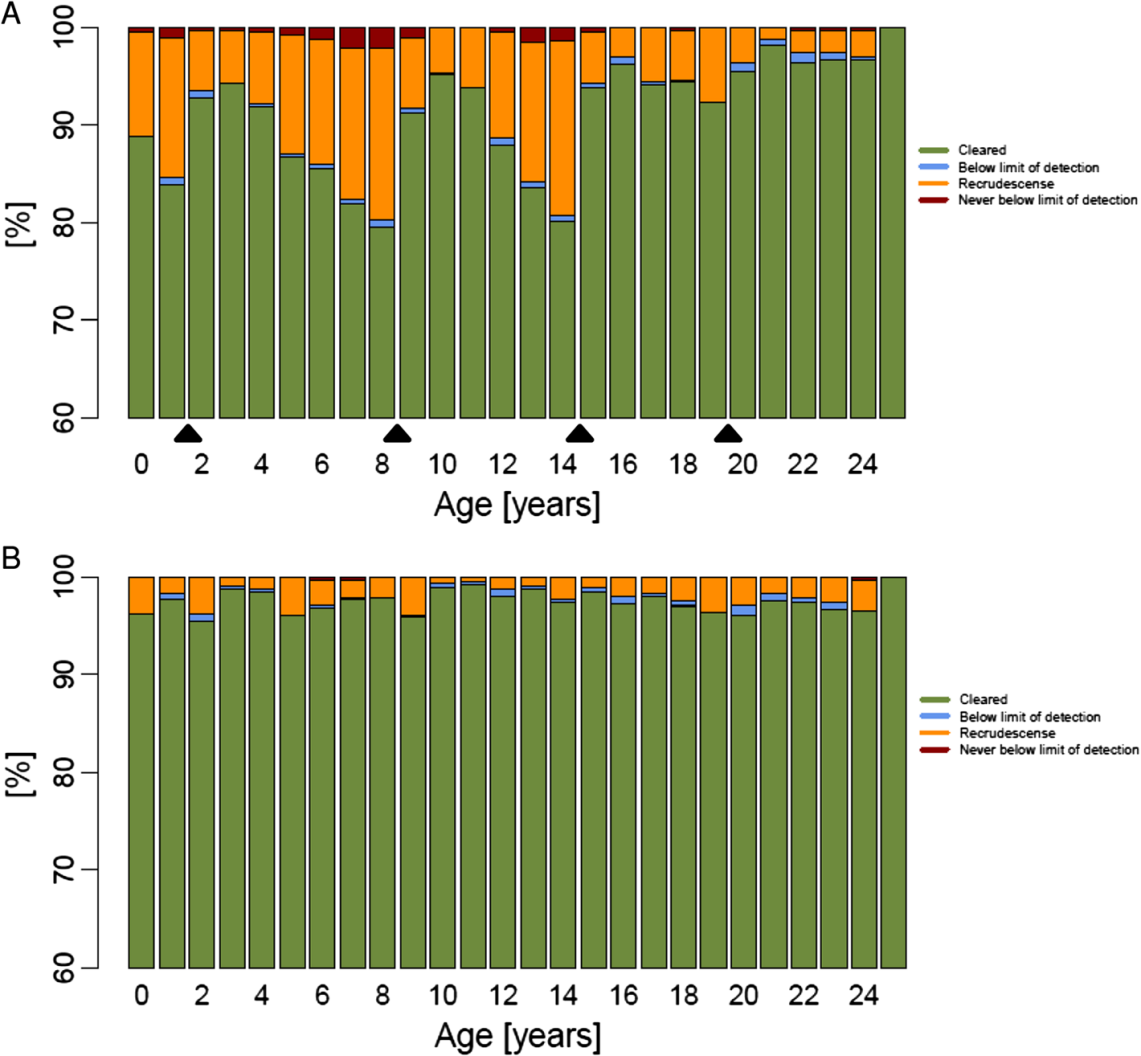
**Predicted dihydroartemisinin-piperaquine treatment outcome per age group (in years) in an African population of 10,000 individuals dosed by (A) age or (B) weight (i e, patients receive one dose daily over three days given at 0 h, 24 h and 48 h).** Black triangles indicate the cut-off points of the age-based dosing bands.

Treatment outcome categories for each age group (in years) were plotted in stacked bar charts (for illustrative purposes examples from African population are presented). Age-based dosing regimen for DHA-PPQ (Figure 3) showed that the percentages of patients cured along the age groups exhibited a ‘saw-tooth’ pattern, i e, the highest cure rates could were seen in individuals at the lower cut-off of each age band and within the same age band cure rates decreased with increasing age. This reflects the fact that younger individuals in a band tend to weigh less and therefore receive a higher mg/kg dose and consequently have higher chance to fully clear all parasites. Conversely, individuals towards the upper cut-off in an age-band are more likely to receive lower mg/kg doses. In older adolescents and adults this trend flattens because once individuals have reached their adult stature, weight is more likely associated with nutritional status than age.

### Adherence scenarios

**Figure 4:**
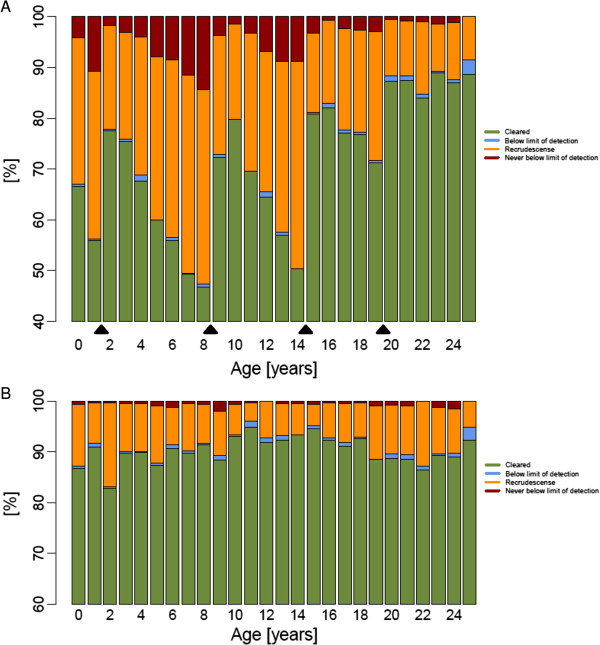
**Predicted dihydroartemisinin-piperaquine treatment outcome per age group (in years) in an African population of 10,000 individuals dosed by (A) age or (B) weight and last dose skipped (i e, patients receive one dose daily over two days given at 0 h and 24 h).** Black triangles indicate the cut-off points of the age-based dosing bands.

Various patterns of non-adherence were investigated to reflect the patterns of non-adherence reported in the literature [40-43]. These typically involve skipping one or several doses. The scenarios investigated for AM-LF were (i) skipping the fifth and/or the sixth doses(s) (ii) skipping the third to sixth dose inclusive. The scenarios for DHA-PPQ were skipping the second and/or third dose. Cure rates for AM-LF stayed as high as 99% when at least four doses were given regardless of region (Table 5) and only a reduction of the total dose to one third reduced cure rates to around 95%. For the age-base dosing regimen of DHA-PPQ cure rates dropped down to 70% when either the second or third dose was skipped (Figure 4A) and down to around 30% when only a single dose of DHA-PPQ was administered (Table 6). The weight-based dosing regimen performed remarkably better with cure rates at around 90% when either the second or third dose was skipped (Figure 4B) or around 50% when only a single dose of DHA-PPQ was administered (Table 6).

Results suggest that AM-LF is less susceptible to non-adherence than DHA-PPQ. Skipping one or two days of treatment of DHA-PPQ (equivalent to one or two doses, respectively) affected treatment outcome to a much greater extent than skipping one or two days of treatment of AM-LF (equivalent to two or four doses, respectively). Skipping the last day of AM-LF treatment, resulted in very high predicted cure rates, i e, 99%. In contrast, skipping the last day of DHA-PPQ gave predicted cure rates of 70% or 90% depending on whether patients were dosed according to age or weight, respectively (Tables 5 and 6). If the last two days (i e, last four doses) of AM-LF were skipped, cure rates dropped down to around 95%, the threshold that would lead to a policy change of first-line therapy [44]. In contrast, skipping the last two days of DHA-PPQ treatment resulted in failure rates of 28% or 50% depending on whether patients were dosed according to age or weight, respectively (Tables 5 and 6). Similarly, the saw-tooth pattern observed for the stacked bar charts of cure rates in every age group became more prominent at lower adherence levels of DHA-PPQ (Figure 4).

Another non-adherence scenario assumed that each individual ‘forgot’ to take a dose and then continued with the regular dosing schedule until the full course was finished on the fourth day. AM-LF was administered at 0, 8, 36, 48, 60, and 72 hours, i e, the third, fourth, fifth and sixth dose were delayed by 12 hours. DHA-PPQ was administered at 0, 48 and 72 hours, resulting in a delay of the second and third dose by 24 hours. This delay and the resulting expansion of the treatment duration to four days did not affect treatment outcome at day 63 (Tables 5 and 6).

To simulate poor adherence to instructions on food intake with ACT, a scenario where patients would receive a full course of AM but only half the respective amount of LF contained in the coformulated tablet at every dose intake (equivalent to decreased bio-availability by 52% due to LF intake without fat [45]) was simulated. However, decreased bio-availability due to fasting when taking AM-LF did not affect treatment outcome (Table 5).

### Drug resistance scenarios

**Figure 5:**
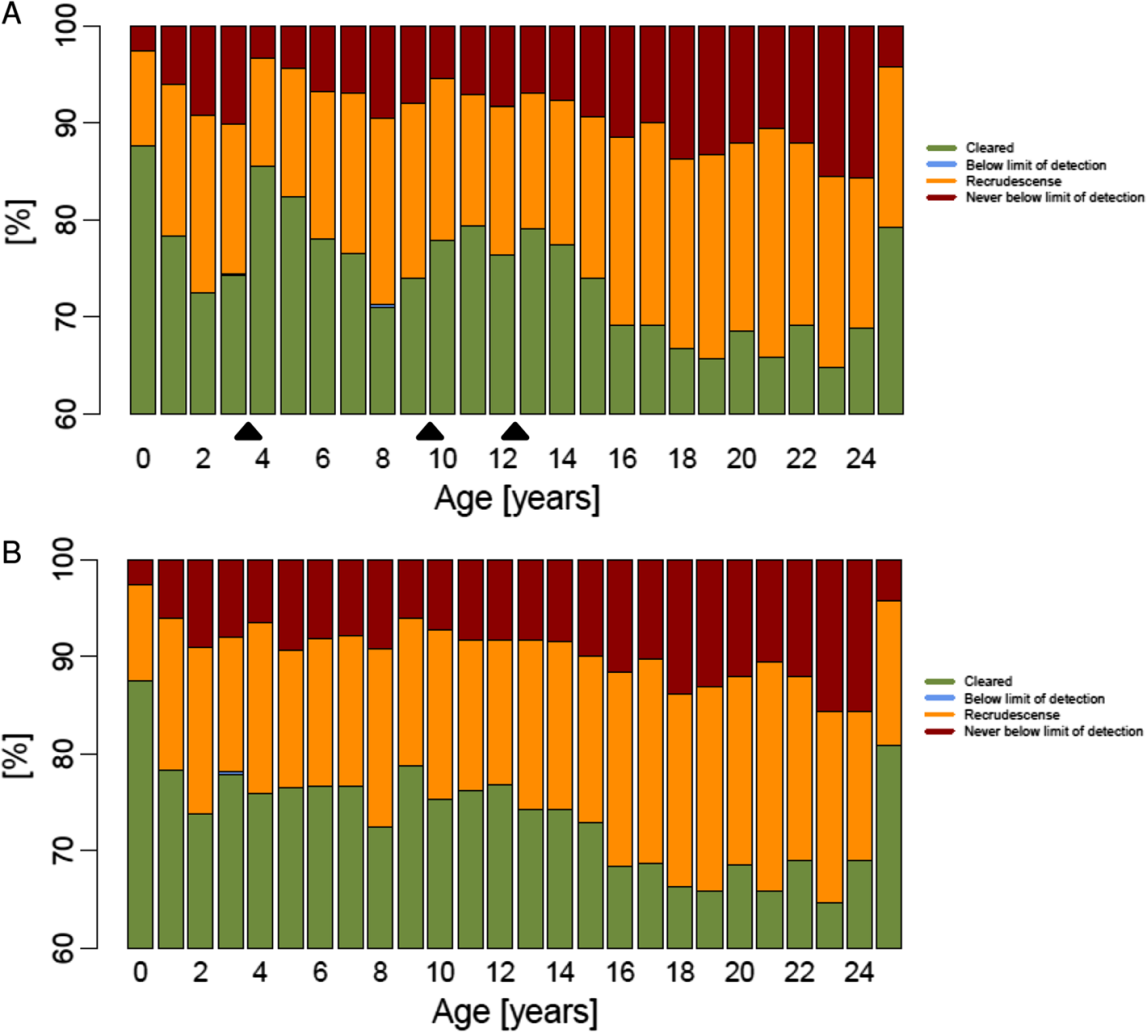
**Predicted artemether-lumefantrine treatment outcome per age group (in years) in an African population of 10,000 individuals dosed by (A) age or (B) weight when the IC**_**50 **_**of artemether and dihydroartemisinin are 10-fold increased and the IC**_**50 **_**of lumefantrine is 50-fold increased.** Black triangles indicate the cut-off points of the age-based dosing bands.

The impact of resistance on treatment outcome was investigated by assessing a full course of AM-LF with IC_50_ values for AM and DHA ten-fold increased and for LF 50-fold increased. These increases in IC_50_ values were chosen because previous analyses showed that this would lead to an increase in drug failure rates to around 10% (Figure 2 in [3]), equal to the threshold recommended by the WHO for a change of treatment regimen [30]. In the simulations cure rates dropped down to 75% (Table 5 and Figure 5). The age distribution of treatment failures when age-based dosing was applied shows that treatment failure is most likely in individuals at the upper thresholds of each age-band because they are more likely to get a lower mg/kg dose.

### Time to recrudescence

The time to recrudescence (Tables 7 and 8) seemed largely consistent for each ACT. Those individuals who fail treatment with a full course of AM-LF fail early because the randomly assigned PK/PD parameters do not allow full clearance of parasites. Those who fail treatment when the total dose of AM-LF is decreased fail rather late (because the period with drug concentrations above the minimal inhibitory concentration [MIC] is shorter with an incomplete regimen due to the reduced area under the drug concentration/time curve [AUC]), thereby paradoxically increasing the mean time to recrudescence. The more doses that are missed the shorter the mean time to recrudescence. There was also a clear decreased in time to recrudescence for AM-LF when the IC_50_ for AM, DHA and LF were increased. For DHA-PPQ, there was a slight decrease in mean time to recrudescence when the total dose was decreased.

**Table 7:** **Predicted time to recrudescence (in days) for artemether-lumefantrine (AM-LF) in populations of 10,000 individuals for different treatment scenarios group by dosing regimen (according to Table **2**) and geographical region**

**Treatment scenario**		**Age-based dosing regimen**				**Weight-based dosing regimen**			
Full course, i e, twice daily over three days at 0, 8, 24, 36, 48 and 60 hours	Min.	14	12	14	11	14	12	14	11
	1st Qu.	22	19	24	18	22	19	24	18
	Median	29	27	31	28	29	28	30	28
	Mean	33	31	34	30	31	31	31	29
	3rd Qu.	46	46	48	44	41	43	38	38
	Max.	57	53	58	52	57	53	56	52
Fifth dose missed	Min.	10	8	10	8	10	8	10	8
	1st Qu.	17	14	19	20	17	17	19	20
	Median	30	28	30	35	25	28	28	36
	Mean	30	30	31	32	30	31	32	33
	3rd Qu.	44	45	46	43	46	46	47	43
	Max.	54	59	55	57	54	59	57	57
Sixth dose missed	Min.	10	8	10	8	10	8	10	8
	1st Qu.	17	14	19	20	17	17	19	20
	Median	30	28	30	35	25	28	28	36
	Mean	30	30	31	32	30	31	32	33
	3rd Qu.	44	44	45	43	46	46	46	43
	Max.	54	59	55	57	54	59	56	57
Fifth and sixth dose missed	Min.	6	5	7	5	6	5	7	5
	1st Qu.	25	28	22	27	19	28	22	27
	Median	35	38	33	38	37	37	36	38
	Mean	33	35	33	36	33	34	33	35
	3rd Qu.	43	45	43	45	43	45	44	45
	Max.	62	61	62	60	62	61	59	60
Third, fourth, fifth and sixth dose missed	Min.	3	5	5	3	3	4	3	3
	1st Qu.	19	21	23	21	19	18	18	18
	Median	26	30	31	29	25	25	25	24
	Mean	27	30	31	30	26	26	26	25
	3rd Qu.	34	39	39	38	34	33	34	32
	Max.	61	61	63	63	61	63	61	63
Third, fourth, fifth and sixth dose delayed by 12 hours	Min.	14	12	14	11	14	12	14	11
	1st Qu.	22	19	24	18	22	18	24	18
	Median	29	26	31	29	29	27	30	28
	Mean	33	30	34	30	32	29	31	29
	3rd Qu.	46	40	48	44	41	38	38	38
	Max.	57	54	58	52	57	54	56	52
Increased IC_50_ by 50-fold for LF and 10-fold for AM and DHA	Min.	5	5	5	5	5	5	5	5
	1st Qu.	15	14	15	14	15	14	14	14
	Median	20	20	20	20	20	20	20	20
	Mean	21	21	21	20	21	21	21	20
	3rd Qu.	27	27	27	26	26	26	26	26
	Max.	51	57	51	62	54	57	55	55
Administered without food, i e, 50% lower dose for LF	Min.	11	12	14	11	11	9	12	8
	1st Qu.	20	19	24	18	20	19	15	18
	Median	25	27	31	28	23	28	24	29
	Mean	28	31	34	30	27	29	27	30
	3rd Qu.	39	46	48	44	34	40	34	41
	Max.	52	53	58	52	52	48	52	49

DHA: dihydroartemisinin; IC_50_: Concentration producing half the desired effect; Min.: minimum; Qu.: quartile; Max.: maximum.

**Table 8:** **Predicted time to recrudescence (in days) for dihydroartemisinin-piperaquine (DHA-PPQ) in populations of 10,000 individuals for different treatment scenarios group by dosing regimen (according to Table **2**) and geographical region**

**Treatment scenario**		**Age-based dosing regimen**				**Weight-based dosing regimen**			
Full course, i e, once daily over three days at 0, 24 and 48 hours	Min.	5	5	5	5	7	6	5	6
	1st Qu.	18	17	18	19	25	25	25	26
	Median	25	25	26	27	34	34	34	32
	Mean	27	27	27	28	36	35	35	33
	3rd Qu.	35	35	35	36	47	45	46	41
	Max.	63	62	63	63	63	63	63	63
Second dose missed	Min.	5	5	5	5	5	5	5	5
	1st Qu.	13	14	14	14	21	21	22	21
	Median	23	23	22	23	29	29	29	29
	Mean	24	24	24	25	31	30	31	30
	3rd Qu.	33	32	32	33	40	38	39	37
	Max.	63	63	63	63	62	63	63	63
Third dose missed	Min.	4	4	4	4	4	4	4	4
	1st Qu.	12	13	13	13	21	20	21	20
	Median	22	22	21	22	29	29	29	29
	Mean	23	23	23	23	30	29	30	29
	3rd Qu.	31	31	31	32	39	38	39	37
	Max.	63	63	63	63	63	63	63	63
Second and third dose missed	Min.	3	3	3	3	3	3	3	3
	1st Qu.	7	7	7	7	12	11	12	11
	Median	15	15	15	16	21	21	22	21
	Mean	19	18	18	19	23	22	23	22
	3rd Qu.	28	27	28	28	32	31	32	31
	Max.	63	63	63	63	63	63	63	63
Second and third dose delayed by 24 hours	Min.	6	6	6	6	7	6	7	8
	1st Qu.	18	18	19	20	25	25	24	25
	Median	26	27	27	28	34	34	34	33
	Mean	27	28	28	29	36	35	35	33
	3rd Qu.	35	36	36	37	48	45	47	41
	Max.	63	63	63	63	63	63	63	63

Min.: minimum; Qu.: quartile; Max.: maximum.

## Discussion

This work identified a PK/PD modelling tool that could support a more standardized approach towards optimizing the programmatic delivery of anti-malarials based on weight- or age-bands and exploring their population level performance in real-life settings. To illustrate this approach, treatment outcome for AM-LF and DHA-PPQ at population level was compared and the potential impact of programmatic factors, including regionally optimized age-based dosing regimens, poor patient adherence and drug resistance on treatment outcome were investigated. Predicted cure rates for both treatments were very high in fully adherent patients. The proportion of individuals that cleared all parasites after treatment with AM-LF was predicted to be more than 99% regardless of region or assessed dosing regimen provided that at least the first four of the six doses were taken. This is in line with a study using a four-dose regimen for AM-LF [21] that reported high cure rates in an area in the absence of multidrug-resistant parasites. DHA-PPQ cure rates in this study were around 97% for the weight-based dosing regimen in fully adherent patients. This is consistent with a pooled analysis that reported polymerase chain reaction (PCR)-corrected cure rates of 98.7% for DHA-PPQ [46]. Thus the basic model seems capable of reproducing what occurs in controlled settings typical of clinical studies, increasing confidence in the model’s ability to accurately predict the impact of important programmatic factors (such as poor compliance and the threat posed by drug resistance) that are more difficult to quantify and use for direct comparison in the validation process of the model. Interestingly, results suggest that the assessed optimized age-based dosing regimens would achieve similar average cure rates for an entire population compared to weight-based dosing regimens in the case of AM-LF but would incur unacceptably high failure rates of around 10% in the case for DHA-PPQ. These findings suggest that AM-LF is more robust than DHA-PPQ in this respect and that the ability of DHA-PPQ to clear infections is hampered by a suboptimal ratio of DHA:PPQ (1:8 mg) and associated low DHA dose. In comparison, the AM-LF ratio of 1:5 mg in the fixed-dose combination exceeds the optimal ratio based on the WHO recommended target dose of 1:7 mg [30]. A 1:4.5 mg DHA:PPQ ratio might be preferable as this is the ratio of the WHO recommended target intake doses for the two components, i e, 4 mg/kg of DHA and 18 mg/kg of PPQ [30]. However, the WHO artemisinin dose recommendation is based on studies of artesunate which has a higher molecular weight than DHA (384.4 *versus* 284.4 mg/mmol, respectively). Consequently, a target intake dose of 3 mg/kg DHA would be equivalent to 4 mg/kg artesunate, suggesting an alternative plausible 1:6 mg ratio to achieve 3 mg/kg of DHA and 18 mg/kg of PPQ. The narrow therapeutic dose range of PPQ compared to DHA (16–26 mg/kg *versus* 2–10 mg/kg per dose [30], i e, a therapeutic index (TI; the ratio of the maximum to minimum recommended therapeutic intake dose in mg/kg) of 1.6 *versus* 5.0, respectively) means PPQ drives the weight or age cut-offs in DHA-PPQ regimens, leaving DHA at a high risk of under-dosing. The average dose for DHA was 2.5 mg/kg and 19.7 mg/kg for PPQ (63 and 109% of the recommended target dose [30]) in 10,000 predicted individuals based on the global weight-for-age distribution. These results are similar to the findings from a pooled analysis by WWARN on DHA-PPQ where 19.6% of patients received a total dose of DHA over three days of less than 6 mg/kg (the lower limit for DHA recommended by the WHO [30]), 20.3% of patients received less than the WHO recommended lower limit of PPQ (48 mg/kg total dose over three days). The corresponding percentages in the simulation presented here were 19.89% for each drug. The WWARN analysis identified that children aged 1–5 years were at greatest risk of receiving a sub-therapeutic doses compared to all other age groups [39]. The simulations presented here also found children under the age of 5 years at particular high-risk, i e, 44.13% with sub-therapeutic doses. In the WWARN study the dose of DHA-PPQ was found to be a significant predictor of parasite positivity on Day 3, and the dose of PPQ was a significant predictor of recrudescence (Table 6 and Figure 4 in [39]). Individuals with low DHA-PPQ were, as expected, more likely to have detectable parasites (> 10^8^) at Day 42 (n = 182) and the results obtained here were extremely similar to those reported in the WWARN study (Figure 2D here *versus* Figure 4 in [39], noting that the difference in x-axis scale arises because Figure 2 here is dose per administration so has to be multiplied by three for total doses). The one difference is that predicted failure rates in the simulations became very low at high drug levels whereas the WWARN tended to plateau off at around 2% even at high drug dosages. Figure 2 was produced assuming full adherence so it is likely that some failures occurred in the WWARN meta-analysis even at high drug dosages due to poor adherence. Due to the low number of individuals with more than 10^8^ parasites at Day 3 (n = 44), no clear trend in dose and parasite positivity was found for Day 3.

In practice, full adherence to anti-malarial drug regimens can be less than 50% in ‘real life’ situations [47]. Reasons for non-adherence to anti-malarials are manifold, including adverse events (AEs) [48], rapid clinical recovery [42,48-52], misunderstanding of instructions [50,52] and, especially in children, difficulties in administration [48,50-52] and fear from over-dosing (‘too many tablets’) [50,52]. Fixed-dose combinations, user-friendly packaging and paediatric formulations adopted for newly developed anti-malarials may help improve adherence, but a certain level of non-adherence is inevitable and programmatic deployment policies need to mitigate its impact. Simulations suggested that delaying a dose did not alter treatment outcome or time of recrudescence (Tables 5, 6, 7 and 8) suggesting that this form of non-adherence does not threaten drug effectiveness when only a single parasite population is present. Generally, when the right mg/kg dose is taken, delaying one dose of the regimen did not lead to significant levels of failures, whereas decreasing the total dose did. These results might change in a more complex model of several parasite populations with different levels of sensitivity to the drug.

A recent study in Papua New Guinea [53] reported low effectiveness of AM-LF. While this could be explained by a number of different factors, this study investigated whether this observation could be simply due to poor bio-availability of LF if it is not taken with fatty food. Cure rates for AM-LF remained above 99% even when the bio-availability of LF was decreased by around 50%. In this case, the presented model may be a helpful tool in testing hypotheses on low effectiveness in clinical trials as it suggests lack of fatty food may not the reason.

Drug resistance is a long-term concern for all anti-malarials because it can drastically shorten the anti-malarials’ therapeutic life-span. While rarely taken into account, the robustness of a drug and its dosing regimen against increasing drug tolerance of the parasites should be considered when deploying new treatment regimens. The presented modelling tool allows exploring the potential threat posed by drug resistance on treatment outcome of different drug regimens by simply altering IC_50_ value of the parasite population. Previous work used the PK/PD model and allowed patients to acquire new infections during the course of follow-up (Appendix of [54]). The model described in this paper could easily be extended to describe emergence of resistance during therapy by incorporating multiple parasite clones that would then be selected depending on selection pressure posed by the drug treatment.

The authors are not dogmatic about the exact PK/PD method used to predict cure and note there are variations on the *in silico* method; for example, users may want to use one-hour time steps to track stage specificity, reparameterize the models according to their own local PK/PD estimates or investigate different putative regimens. Notably, the model does not include acquired host immunity at present. While this can be incorporated (e g, [13]) it was omitted at this stage as its calibration relies on arbitrary parameters. Drugs must be able to reliably cure patients irrespective of their immune status, so the absence of assumptions on level of immunity will result in the most conservative recommendations. Similarly, further fine-tuning of this method could involve the inclusion of differences in PK parameters in specific subgroups of the population, such as children and pregnant women, if available.

In essence, a methodology is described that provides a more probabilistic approach to optimizing dosing based on PK/PD modelling to estimate treatment outcome according to proposed deployment policies. This tool can support the development process for ACT with low TIs (e g, PPQ or mefloquine), where dosing all patients within the narrow therapeutic dose range will not be feasible using age-based dosing or weight-based dosing regimens that only use four dose categories (e g, avoiding use of complex regimens with tablet fractions). In these cases, deployment decisions on appropriate banding of dosing regimens will be a compromise between optimal dosing accuracy and programmatic feasibility. This study used simple PK/PD modelling and the primary intention is to demonstrate a methodological ‘road map’ capable of addressing important deployment issues. For example, while the lower therapeutic dose for PPQ is defined as 16 mg/kg, this does not mean that those receiving less than 16 mg/kg will inevitably fail treatment and those receiving 16 mg/kg or above will always be cured. Someone receiving 15.9 mg/kg is less likely to fail treatment as someone receiving 10 mg/kg. In reality many factors determine whether or not a patient fails treatment and many ‘under-dosed’ individuals will still be cured because they have relatively drug-sensitive parasites or they metabolize drugs slower and maintain high drug concentrations for longer. The method simply aims to define and capture this complexity to support the (so far unregulated) decision-making process to translate the regulatory mg/kg dosing recommendation to programmatic dosing based on weight or age bands.

The obvious next step is to get similar nuanced, probabilistic measures for the risk of toxicity at higher dose exposure. Some AEs are dose-independent and would occur at a constant rate irrespective of the used dosing regimen, e g, in a pooled analysis of individual patient data vomiting or diarrhoea were not correlated with the mg/kg dose of PPQ [39]. Others are dose-dependent and correlated with PK factors, such as the total AUC and the maximum concentration reached (c_max_), e g, the length of the corrected interval between the Q and T wave in the heart’s electrical cycle is positively correlated with halofantrine exposure [55], glucose-6-phosphate dehydrogenase (G6PD) deficiency mediates dose-related toxicity in primaquine [56] and pruritus in sensitive individuals is linked to chloroquine levels [57]. It is of note that the between-subject variability in human PK parameters is typically 30–50% [58]. The PK component of the model estimates factors such as c_max_ and AUC for each patient, allowing replacing the arbitrary upper therapeutic threshold in mg/kg with a more nuanced prediction based on patients’ individual PK. In practice, this is hampered by the lack of reliable, standardized safety data from preclinical and clinical studies. Toxicity data from preclinical studies is mostly unpublished and only accessible to the manufacturer and regulatory authorities. Furthermore, outside clinical trial settings, the relationship between dose and individual AEs is rarely known, reports on accidental over-dosing are scarce, there are no standardized tools to collect or report safety data, and the association to dose or dose exposure levels are rarely reported [59]. If these data were available the methodology would be able to output the proportion of patients in a group receiving toxic doses and the likely degree of their toxicity. For example, this would allow to estimate that x% of patients had ‘mild’ toxicity (perhaps defined as <10% above a critical c_max_). This would allow policy makers to review and balance the risks of toxicity *versus* drug failure based on PK/PD prediction, particularly for anti-malarials with a narrow TI, where it is unavoidable that a substantial proportion of patients will receive a dose outside the established therapeutic dose range.

## Conclusion

This PK/PD modelling approach is a major methodological advance in the rational design of programmatic drug deployment, complementary to the data generated from clinical trials.

## Abbreviations

a: The maximal parasite growth rate; ACT: Artemisinin-based combination therapy; AE: Adverse event; AM: Artemether; AUC: Area under the drug concentration/time curve; cmax: maximum concentration; DHA: Dihydroartemisinin; f(Cd): Effect of the drug(s) administered; f(I): Patients immunity; G6PD: Glucose-6-phosphate dehydrogenase; IC50: Half-maximum inhibitory concentration; k: Elimination rate constant; LF: Lumefantrine; LoD: Limit of detection; MIC: Minimal inhibitory concentration; n: Slope factor; P0: Initial parasite number; PCR: Polymerase chain reaction; PD: Pharmacodynamics; PK: Pharmacokinetics; PPQ: Piperaquine; Pt: Parasite numbers at a given time point *t*; R&D: Research and development; t: Time; TI: Therapeutic index; Vmax: First-order rate constant of parasite killing; Vd: Volume of distribution; WHO: World Health Organization; WWARN: WorldWide Antimalarial Resistance Network; x: Absorption rate constant; z: Conversion rate constant.

## Competing interests

The authors have declared that they have no competing interests.

## Authors’ contributions

IMH and DJT conceived and design the study. All authors were involved in setting up the model or parts of it. EMH was responsible for acquisition of data. All authors were involved in analysis and interpretation of data. EMH, IMH and DJT drafted the manuscript and KK and DJH revised it critically for important intellectual content. All authors have given final approval of the version to be published and agreed to be accountable for all aspects of the work in ensuring that questions related to the accuracy or integrity of any part of the work are appropriately investigated and resolved.
